# Impact of vegetation composition and seasonality on sensitivity of modelled CO_2_ exchange in temperate raised bogs

**DOI:** 10.1038/s41598-024-61229-6

**Published:** 2024-05-14

**Authors:** Claas Voigt, Maren Dubbert, Samuli Launiainen, Philipp Porada, Jan Oestmann, Arndt Piayda

**Affiliations:** 1 Leibniz Centre for Agricultural Landscape Research (ZALF), Eberswalder Straße 84, 15374 Müncheberg, Germany; 2 Thünen Institute of Climate-Smart Agriculture, Bundesallee 65A, 38116 Braunschweig, Germany; 3 Natural Resources Institute Finland (LUKE), Latokartanonkaari 9, 00790 Helsinki, Finland; 4 Institute of Plant Science and Microbiology, Universität Hamburg, Ohnhorststr. 18, 22609 Hamburg, Germany

**Keywords:** Carbon cycle, Ecological modelling, Ecophysiology

## Abstract

Encroachment of vascular plants (VP) in temperate raised bogs, as a consequence of altered hydrological conditions and nutrient input, is widely observed. Effects of such vegetation shift on water and carbon cycles are, however, largely unknown and identification of responsible plant physiological traits is challenging. Process-based modelling offers the opportunity of gaining insights into ecosystem functioning beyond observations, and to infer decisive trait shifts of plant functional groups. We adapted the Soil–Vegetation–Atmosphere Transfer model pyAPES to a temperate raised bog site by calibration against measured peat temperature, water table and surface CO_2_ fluxes. We identified the most important traits determining CO_2_ fluxes by conducting Morris sensitivity analysis (MSA) under changing conditions throughout the year and simulated VP encroachment. We further investigated transferability of results to other sites by extending MSA to parameter ranges derived from literature review. We found highly variable intra-annual plant traits importance determining ecosystem CO_2_ fluxes, but only a partial shift of importance of photosynthetic processes from moss to VP during encroachment. Ecosystem respiration was dominated by peat respiration. Overall, carboxylation rate, base respiration rate and temperature sensitivity (Q_10_) were most important for determining bog CO_2_ balance and parameter ranking was robust even under the extended MSA.

## Introduction

Bog peatlands are characterised by a permanent water saturation and low pH values, hampering organic matter from decomposition^1^ and vascular plants (VP) from encroaching into peat moss (*Sphagnum* ssp.) dominated plant communities^2^. However, due to changing climate and artificial drainage for land use the pristine hydrological regime is often disturbed. For example, lower water levels foster aeration of the peat and hamper capillary water supply for *Sphagnum* mosses, both supporting an encroachment of graminoid and tree species^3^. This phenomenon can be observed in both natural and restored bog peatlands^4,5^.

The presence of VP can have contrasting effects for peatland water and greenhouse gas (GHG) cycling and can even improve conditions for their own expansion. Abundant root penetration of the peat could increase transpiration rate, leading to soil aeration and consequently to a further decrease of the groundwater level, creating a positive feedback loop for establishment of VP^6^. However, it has been shown in other studies, that birch encroachment does not necessarily have an impact on water table^7^, while increased graminoid biomass even diminished evaporation losses by attenuating wind speed at the moss surface^8^ and thus protecting the moss from desiccation^9^. Further, there's evidence, that rather temperature increment seems to be the main driver of graminoid and tree dispersion compared to water regime^6,10^. Some findings suggest, that *Sphagnum* mosses desiccate permanently after extreme heat waves^11^ while others indicate recovered growth and a decrease of tree cover if drought events are followed by a normal water regime^6,12^. Thus, although encroachment is observed also in natural raised bogs, restored bogs are probably more vulnerable due to disturbed hydrological regime and potentially insufficient rewetting measures and consequently, lower ability for recovering.

In a modelling study, Heijmans et al.^10^ reported a negative relationship between VP expansion and peatland carbon accumulation. The presence of VP seems to prime microbial decomposition of organic matter, resulting in increased losses of carbon due to respiration or in dissolved form due to destabilization of organic matter^13,14^. However, short-term observations show that removal of VP resulted in decreased net CO_2_ uptake by 50% during vegetation period in two investigated sites at different elevations^13^. Many research papers focus on how GHG emissions respond to changing water tables^15,16^ or how vegetation composition is affecting biodiversity^17,18^. Due to the large amount of stored carbon in peatlands^1^, even small changes in climatic or hydrological conditions or vegetation composition can have significant impacts on the global carbon balance. When loss of peat carbon due to encroachment exceeds the enhanced sequestration by VP photosynthesis, the bog might turn from a sink into a source of carbon and thus current efforts of peatland protection and restoration for emission mitigation will be diminished. As shown above, implications of VP encroachment on water and carbon balances in bogs require more research, especially regarding predicted future climate conditions. There's also a need to understand physiological and physical key parameters of raised bogs responsible for alterations of the carbon cycle following restoration measures.

Conducting empirical studies to investigate these key parameters impacting carbon cycling would require years or even decades of measurements. Utilizing Soil–Vegetation–Atmosphere Transfer (SVAT) models is an effective tool to get insights into relevant physical and ecological processes and infer crucial parameters from flux simulations. A review of process-based models used for peatland carbon and water balance simulations^19^ showed strong focus on northern peatlands and less on temperate ones. Of the 45 investigated models in that study, only three models specifically address eco-hydrology. Further, only 18 of them consider peatland specific properties such as microtopography, shallow water tables or peat accumulation and there's a large variation regarding temporal and spatial scales. Consequently, using an existing process-based peatland model for simulating CO_2_ fluxes in temperate bogs that (i) represents eco-hydrological processes (ii) at local scale in temperate climate and (iii) in high temporal resolution, will most likely require some amount of structural model adaptation.

Here, we applied the multi-layer SVAT model pyAPES^20,21^, developed and tested under boreal conditions, to a temperate raised bog with shallow water table, peat properties and vegetation composition. We calibrated the model using soil temperature (T_s_), water table dept (WTD) and CO_2_ flux data^22^. pyAPES consists of sub-models for canopy and soil processes. Within the canopy sub-model, a separate moss layer is described that can be parametrized with specific traits measured in-situ or obtained from literature. This includes physical properties such as moss height (h_m_) and bulk density (ρ_m_), water retention characteristics, and photosynthetic traits^23,24^. Further, the model provides the opportunity to include vascular plants such as graminoids and tree species, characterised by a multi layered leaf area density profile and physical and physiological traits. As the model includes feedbacks between the multi-layer vegetation, microclimate and soil hydrology, pyAPES can partition fluxes originating from soil, moss and VP under changing environmental conditions.

The overall objective of this study is to identify the main eco-physiological traits of the two functional groups (*Sphagnum* moss and VP grass) and peat properties that control ecosystem level CO_2_ uptake (gross primary production, GPP) and emissions (ecosystem respiration, R_eco_), as well as their dynamics caused by environmental conditions. To achieve this goal, we address the following research questions:

I. Which photosynthetic and respiratory parameters (traits) are most crucial for modelling CO_2_ fluxes in a temperate raised bog?
II. Is the observed relative importance of parameters/processes affected by seasonal variability in the environmental conditions (especially T_s_ and WTD)?
III. Does simulated VP encroachment (increasing leaf area index and decreasing *Sphagnum* dry mass) alter the importance of functional groups for CO_2_ balances?

The results will contribute to better understand the interactions between plants and soil in raised bogs and how these interactions regulate the release and accumulation of carbon. Further, our investigation will give insights about pyAPES' performance in modelling CO_2_ exchange and hydrology of raised bogs.

## Results

### Sensitivity of annual fluxes

**Figure 1:**
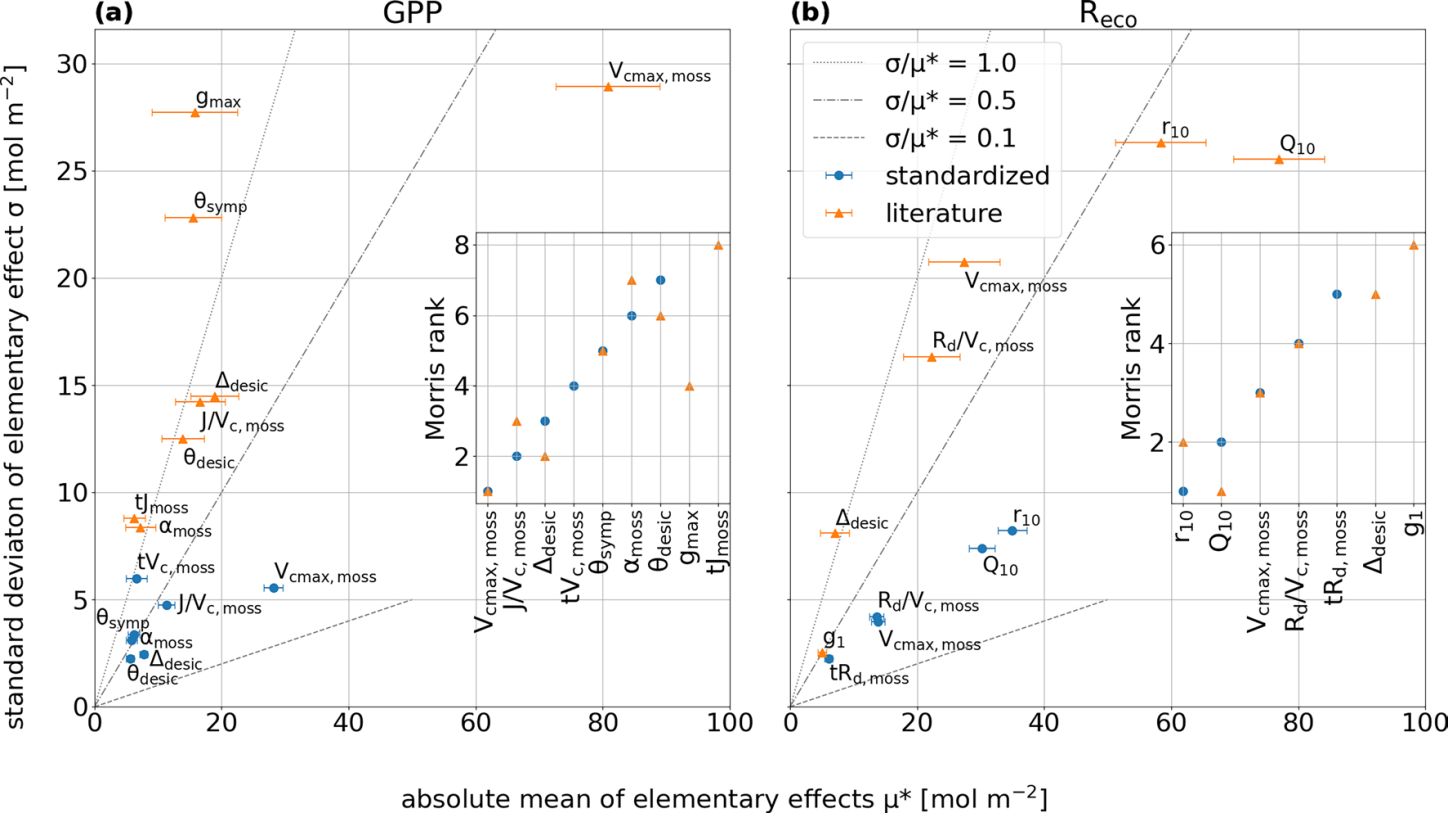
Average sensitivities of annual balances of (a) GPP and (b) R_eco_ to important model parameters (μ* > 5 mol m-2 yr^-1^) at standardized (± 30% variation; blue dots) and literature boundaries (orange triangles). Inset plots show ranking of variables for both boundary conditions and thus display a change in ranking (ranking 1 = most important). Dashed/dotted grey lines representing isolines of the ratio of standard deviation (σ) and absolute mean (μ*) of the elementary effects. At a high ratio, the respective parameter has likely a non-linear effect and/or is involved in interactions with other parameters.

We found only minor changes in parameter ranking by extending the boundaries of Morris sensitivity analysis (MSA), either regarding GPP or R_eco_. However, extending boundaries for MSA enlarged the amplitude of all parameters. Figure 1 shows absolute mean (μ*) and standard deviation (σ) of EE. While dominating parameters that affected GPP annual sums were moss related (Fig. 1a), the highest impact on R_eco_ annual sums was forced from soil parameters (Fig. 1b).

In case of the standardized ranking (± 30% boundaries), overall seven of total 22 parameters were identified to be important (μ* > 5 mol m^−2^ year^−1^) in affecting GPP annual sums. Parameters that are included in photosynthesis processes directly (Farquhar model in pyAPES) play a major role compared to moisture, temperature or radiation related traits. Maximum moss carboxylation velocity (V_cmax,moss_) was clearly found to be the most important parameter, followed by its ratio to maximum transport rate of electrons (J/V_c,moss_) and desiccation induced reduction in photosynthetic capacity (Δ_desic_). The last four parameters that affected GPP most shared the same rank as their confidence intervals overlapped. No parameters of VP appeared significant for GPP of the natural bog, likely due to the low VP leaf-area. The highest rank of this plant functional group was eight, shared by V_cmax,vas_ and J/V_c,vas_. Annual balances of R_eco_ were most sensitive to peat respiration base rate and temperature sensitivity (r_10_ and Q_10_), followed by V_cmax,moss_ and its ratio to dark respiration rate (R_d_/V_c,moss_) and, finally, temperature dependency of dark respiration (tR_d,moss_). Similar to GPP, no parameters of VP were considered important for R_eco_ balances.

By expanding parameter boundaries of MSA from the previously used ± 30% to boundaries based in a literature review, the amplitude of all parameters increased. In case of GPP, two more parameters became important: the air-to-chloroplast conductance for CO_2_ when external water has evaporated (g_max_) and the temperature sensitivity of electron-transport limited photosynthesis rate (tJ_moss_). On the other hand, the temperature sensitivity of RuBisCO limited photosynthesis rate (tV_c,moss_) was no longer part of the group of most important parameters. Regarding R_eco_, only tR_d_ was no longer found to be important, while Δ_desic_ and VP parameter g_1_ (stomatal slope) became important here. Overall, all parameters showing a high μ* (V_cmax,moss_ and J/V_c,moss_ for GPP; r_10_, Q_10_, V_cmax,moss_ and R_d_/V_c,moss_ for R_eco_) did not shift more than one rank. Increased μ* and σ values were found, as well as wider confidence intervals (CI) and coefficients of variation (cv, σ/μ*) for all shown parameters (Fig. 1) compared to standardized boundaries for both, GPP and R_eco_ fluxes.

### Dependence of flux sensitivity on environmental conditions

**Figure 2:**
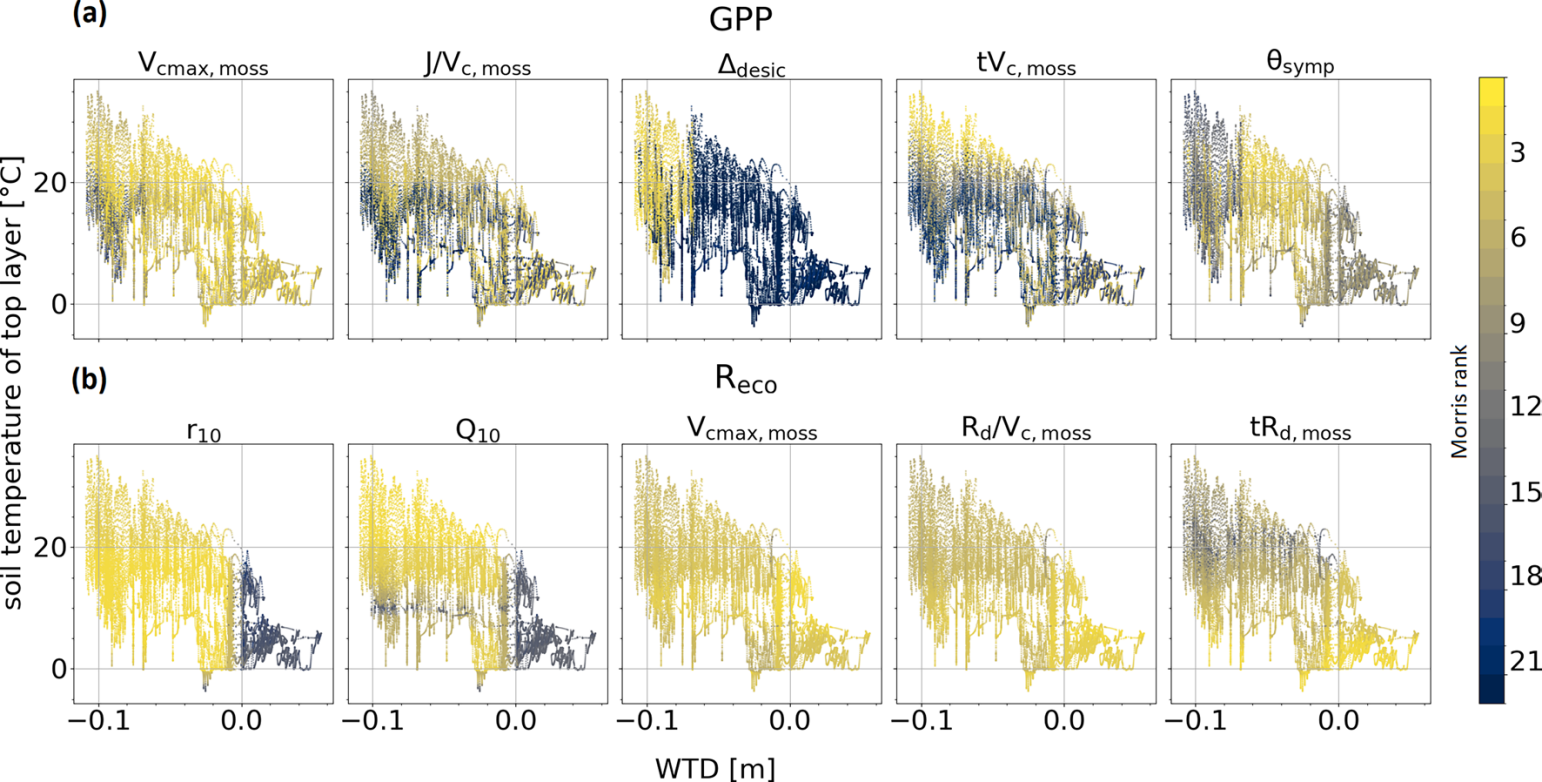
Impact of water table depth (WTD) and soil temperature (T_s_) of the top soil layer (1 cm depth) to Morris ranking of the five most important model parameters (according to absolute mean without considering confidence intervals) of (a) GPP and (b) R_eco_. Negative signs of WTD indicate water table is below surface.

With regard to research question II, we found an effect of WTD on both, GPP and R_eco_ flux sensitivity for nearly all parameters (Fig. 2), and the parameter rankings for GPP were the most sensitive to changing environmental conditions.

GPP was much more susceptible to rate of decrease of photosynthetic capacity in dry moss (Δ_desic_) at deeper WTD lower than – 5 cm (Fig. 2a). In contrast, maximum symplast water content (θ_symp_) was more relevant for GPP at water levels close to surface and showed decreasing importance with both declining and increasing WTD, but with a stronger intensity in the direction of deeper WTD. Lower sensitivity values for V_cmax,moss_ only appeared, if water level and temperature were low at the same time. Increased sensitivity to J/V_c,moss_ ratio was found with rising WTD compared to deeper WTD, given similar temperature levels. Temperature optimum of V_cmax,moss_ (tV_c,moss_) showed high and low sensitivities mixed over the whole range of WTD. However, at T_s_ above 25 °C only very high ranks occurred. For R_eco_ we found a strongly declining influence of r_10_ and Q_10_ with water table above soil surface (Fig. 2b). V_cmax,moss_ and R_d_/V_c,moss_ respectively showed slightly increasing ranks with shallower WTD while there was no clear pattern for the other parameters.

Temperature also affected flux sensitivities. Regarding tV_c,moss_ there was a strong drop in its relevance for GPP with decreasing temperatures, but only when WTD was close to surface (i.e. no water limitations). J/V_c,moss_ was declining more gradually with temperature for the whole range of WTD. Despite a small temperature dependency for V_cmax,moss_ at deeper WTD, other important parameters showed no clear pattern. Most of the important parameters for R_eco_ showed a relationship to T_s_. While the effect was minor, stronger impact of T_s_ could be shown for tR_d,moss_ with lower ranks by increasing temperatures. The only parameter that increases ranking with increasing T_s_, given lower WTD, was Q_10_.

To quantify these relationships, linear regression was used. For both GPP and R_eco_, the results showed strong impact of WTD and T_s_ on average elementary effects μ* for all shown parameters (Table 1), illustrating clearly changing absolute sensitivities (μ*) with varying water table and soil temperature. Except tR_d,moss_, all regression models showed a better fit (R^2^) to R_eco_ values than to GPP, confirming less variation in ranking compared to GPP. Nearly all parameters responded with increasing μ* to increasing WTD and T_s_, respectively. Only Δ_desic_ was found to affect GPP fluxes less with higher water table. Further, the decreased impact of tR_d,moss_ with rising soil temperature was confirmed. In all cases varying WTD by one unit affected fluxes much more than varying T_s_. Thus, in average CO_2_ fluxes became more sensitive to all presented parameters (Fig. 2, Table 1) with increasing WTD and T_s,_ except Δ_desic_ and tR_d_. Relative standard errors were low for all coefficients, resulting in a narrow CI_95_ and thus a robust estimate.

**Table 1:** Estimate and relative standard error of μ* (SEM_rel_), upper and lower boundaries of 95% confidence intervals (CI_95_) of coefficients b and c and R^2^ of linear regression model of the form μ* = a + b × WTD + c × T_s_ for the five most important target variables of annual balances.

	b (WTD)				c (T_s_)				R^2^
	Estimate	SEM_rel_	CI_95_		Estimate	SEM_rel_	CI_95_		
			Lower	Upper			Lower	Upper	
GPP									
V_cmax_	4.89	0.04	4.49	5.28	0.102	0.01	0.100	0.104	0.28
J/V_c_	0.81	0.10	0.65	0.97	0.035	0.01	0.034	0.036	0.25
Δ_desic_	− 2.92	0.04	− 3.15	− 2.68	0.038	0.02	0.037	0.039	0.25
tV_c_	3.01	0.05	2.71	3.31	0.078	0.01	0.077	0.080	0.30
θ_symp_	3.38	0.04	3.13	3.63	0.056	0.01	0.054	0.057	0.22
Reco									
r_10_	3.75	0.04	3.48	4.02	0.145	0.01	0.143	0.146	0.66
Q_10_	7.41	0.03	6.95	7.87	0.208	0.01	0.206	0.211	0.56
V_cmax_	1.83	0.01	1.78	1.88	0.026	0.01	0.025	0.026	0.56
R_d_/V_c_	1.77	0.01	1.72	1.82	0.024	0.01	0.024	0.024	0.54
tR_d_	0.56	0.04	0.52	0.60	− 0.003	0.04	− 0.003	− 0.002	0.12

Units are in [m] for WTD and [°C] for T_s_. All shown parameters are related to moss.

### Impact of vegetation composition on flux sensitivities

**Figure 3:**
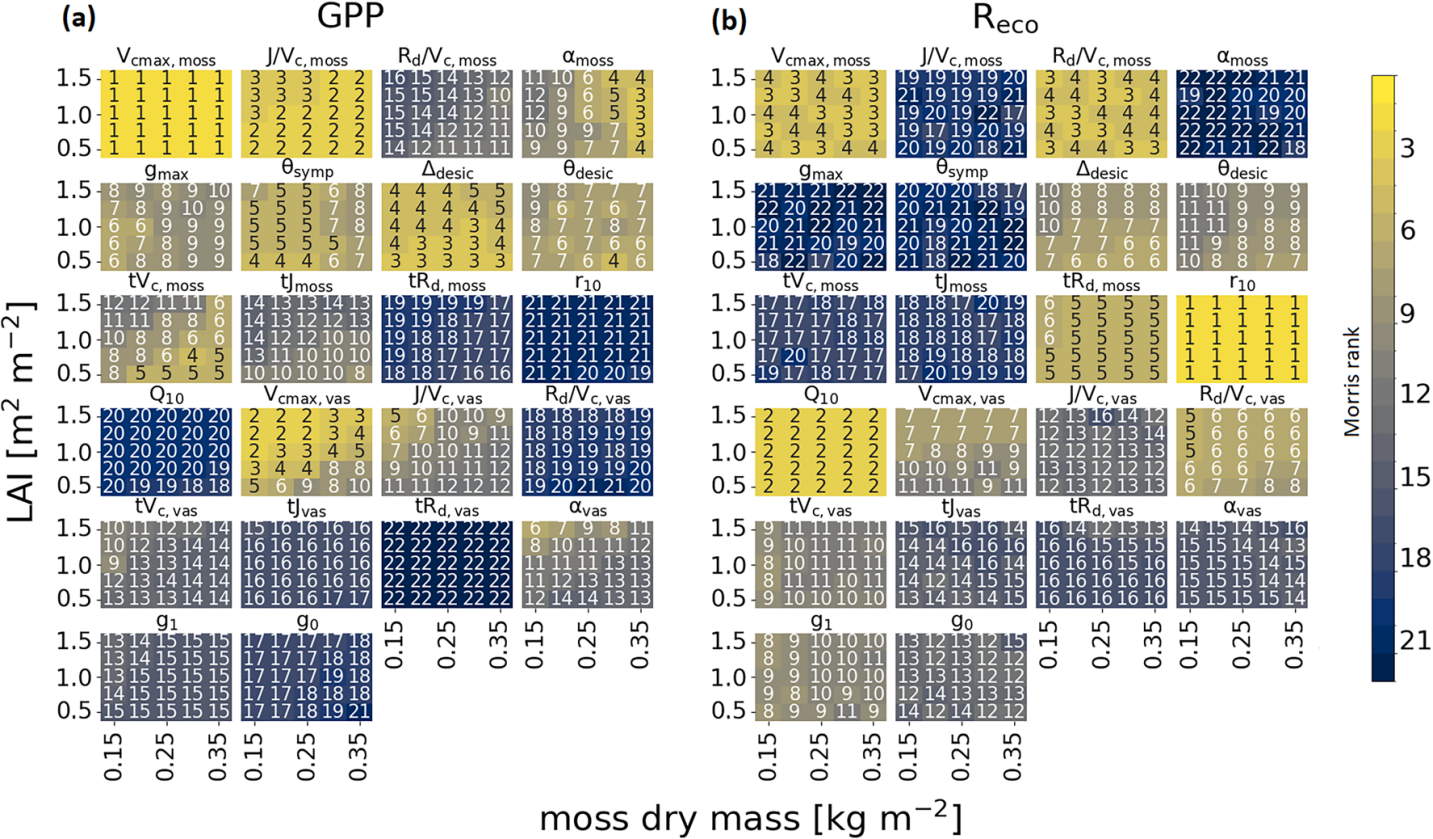
Impact of simulated change in vegetation composition (leaf area index of vascular plants, LAI and moss dry mass, m_dry_) on annual sums of (a) GPP and (b) R_eco_ while moss ground cover was still assumed to be nearly 100 %. Numbers and colors in panels indicate Morris ranking (1/light = most important to 22/dark = less important).

The simulated shift in vegetation composition (research question III) had a clear impact on parameter rankings, more distinct for annual GPP than for R_eco_ (Fig. 3). However, the V_cmax,moss_, r_10_ and Q_10_ remained the most important irrespective of decreasing m_dry_ nor increasing vascular LAI.

The maximum carboxylation rate of vascular plants (V_cmax,vas_) became increasingly more important for GPP with decreasing m_dry_, reaching even rank two when vascular LAI was at its highest. Same pattern, albeit at lower ranking, was observed for several other VP parameters (J/V_c,vas_, quantum yield α_vas_, stomatal conductance parameters g_1_ and g_0_). In contrast, moss parameters affecting GPP increased their importance with enhanced m_dry_ and lower vascular LAI (R_d_/V_c,moss_, α_moss_, tV_c,moss_, tJ_moss_, tR_d,moss_). In case of α and tV_c_, this led to a switch in the order of importance between moss and VP.

Ranking of parameters with respect to annual R_eco_ was again more robust than that of GPP. Both soil parameters (r_10_ and Q_10_) were the dominating parameters followed by V_cmax,moss_ and R_d_/V_c,moss_, both alternating at rank three and four. All of them were unaffected by changed m_dry_ or vascular LAI. However, there were also some parameters that changed importance with simulated encroachment. The role of Δ_desic_ and θ_desic_ both decreased with VP encroachment, whereas the importance of V_cmax,vas_ and R_d_/V_c,vas_ increased. Of the VP parameter V_cmax,vas_ was ranked highest with a value of seven.

## Discussion

The calibration of the model to represent temperate raised bogs was conducted using observations from the Meerkolk site. Since pyAPES was originally developed for canopy covered boreal forest and peatland ecosystems^20,21^, adapting the model to temperate bogs required parameter calibration, mainly concerning peat hydrological properties. The most challenging part was the choice of shape parameters (α and n) of the unimodal van Genuchten–Mualem (vGM) water-retention model. McCarter and Price^25^ showed that peat air-entry potential (α) can be highly variable in the uppermost peat layers consisting on poorly decomposed dead peat forming *Sphagnum*, ranging from 0.4 to 2.6. Values of n used in that study^25^ were more robust (ranging between 1.2 and 1.5) for *Sphagnum* peat of different bulk densities. Price et al.^26^ presented much higher values for weak decomposed *Sphagnum* mosses, which is used in the present study. High α and n values indicate high proportion of macropores and consequently an early entrance of air, well reflecting field observations, as the first soil horizon in Meerkolk was characterized as Von Post degree 1 (very weakly decomposed) and bulk density of living moss was comparably low. Further, higher values of α lead to higher K_unsat_, which may partly compensate the missing shrinkage of the modelled peat profile and thus better reproduce observed WTD. The applied combination of high α (0.8^27^) and n (2.5^26^) describing water retention of the first soil horizon up to − 18 cm in the present study was thus probably necessary to compensate a distinct swelling/shrinkage behaviour as discussed above. Using bimodal vGM curves^28^ to describe dual porosity might thus be beneficial to better capture changes in porosity due to these processes in organic soils^31^.

The current parametrization of moss pF (α = 0.4; n = 2.8) is similar to those reported in Price et al.^26^ (α = 0.5; n = 2.5) for living/undecomposed *Sphagnum*. Given the maximum gravimetric water content of moss layer w_max_ that can be hold against gravity, saturated (θ_sat_) and residual (θ_res_) volumetric water content of moss are computed by using ρ_m_. ρ_m_ varies strongly between *Sphagnum* species and even at the same plot between two years of measurement for the same species. For *Sphagnum papillosum* (dominating Meerkolk species), Bengtsson et al.^29^ reported ρ_m_ comparable to measurements at Meerkolk site (8.3 ± 0.97 kg m^−3^), but about 50% higher one year earlier (13.4 ± 1.63 kg m^−3^). Considering all reported species, ρ_m_ in their study^29^ ranged from 3 to 20 kg m^−3^. Applying Eqs. (6) and (7) using this low ρ_m_, the resulting water retention curve was spanning a very narrow range of θ. This was consequently causing an implausible breakdown of capillary rise in summer 2018, since unsaturated conductivity K_unsat_ decreased too rapidly with θ, although water table was still high (about – 10 cm). Since shrinkage due to desiccation is not implemented, it was necessary to constrain the minimum K_unsat_ of moss to ~ 0.004 mm h^−1^ to enable capillary connection between the peat and the living moss layer throughout the modelling period. Overall, we highly recommend a parametrisation of both, ρ_m_ and w_max_, by measured values, and if possible, measured water retention and conductivity characteristics.

Modelling soil temperature and ground water level was particularly challenging in the hot and dry summer conditions during European 2018 heatwave, while the WTD and soil temperature profile was well simulated during less extreme conditions in 2017 (Fig. 5). This was most likely related to shrinking and swelling of the peat body due to desiccation and rehydration that is currently not implemented in pyAPES. Nijp et al.^30^ found, that in pristine bogs a change in absolute ground water table of – 15 cm can change peat elevation up to – 10 cm. Howie and Hebda^31^ present similar or even higher values for very wet conditions and inundation, comparable to Meerkolk site with shallow WTD. Further, shrinkage reduces the pore size, which tends to lead to higher water retention and an increase in unsaturated conductivity (K_unsat_)^32^, enhancing water supply from deeper soil layers. Including a dynamic soil profile might be more important in temperate compared to boreal latitudes fostering a more accurate description of the moss layer hydrology, even under increasingly extreme climate conditions.

Measurements of CO_2_ fluxes of Oestmann et al.^22^ were conducted from one hour before sunrise until late afternoon. It is common practice to measure GHG fluxes from close before sunrise until afternoon^33–35^, as maximum PAR and soil temperatures are already reached at this time of the day. Measurements over the whole day and even night time would potentially increase quality of the manually conducted model calibration. However, main focus of this paper are outcomes of the subsequent model sensitivity analysis, which does not take into account the observed fluxes explicitly. Thus, the impact of additional flux observations on results on model sensitivity is considered marginal.

It was necessary to scale Farquhar parameters with decreasing m_dry_ for the encroachment simulation, since the model defines these parameters of the moss layer per unit ground area. Despite Scartazza et al.^36^, who found linear relationships of V_cmax_ and J_max_ with leaf mass of European beech (*Fagus sylvatica*) trees, evidence for the nature of this relationship to moss dry mass is missing. Thus, linear scaling was used.

Annual bog GPP was most sensitive to maximum carboxylation rate V_cmax,moss_, followed with greater distance by J/V_c,moss_, Δ_desic_, tV_c,moss_, θ_symp_, α_moss_ and θ_desic_. Thus, under the studied environmental conditions, the bog GPP variability was primarily controlled by moss photosynthetic traits, water holding capacity, as well as the shape of photosynthetic moisture response. The relative importance (ranking) of V_cmax,moss_ was barely affected, neither due to extended boundaries, WTD, T_s_ nor increase of VP LAI. Only temperatures below 20 °C and WTD < − 7.5 cm were reducing its impact. This relation to WTD and T_s_ could be observed for all important parameters, and is probably the joint effect of desiccation reduction and temperature dependant increase of photosynthesis. J_max,moss_ (represented by its ratio J/V_c_) is also determining photosynthesis rate of moss directly. Lower ranks of J_max,moss_ were found, similar to V_cmax,moss_, only at low water tables and temperatures. Sensitivity to J_max_ is thus also declining as maximum electron transport rate is never reached with deficit of both water and light. High temperature, on the other hand, is often associated with high irradiance which is increasing J and attenuating this limitation. Consequently, very low ranks of V_cmax_ and J_max_, respectively could mainly be observed when both, WTD and T_s_ were low. Albeit ranking of V_cmax,moss_ did not change under simulated encroachment, i.e. increasing VP LAI, shading of VP started to decrease absolute sensitivity (μ*) of moss V_cmax_ and J_max_ (data not shown). This is not reflected in relative importance (ranking) since μ* of moss traits was still higher than those of VP.

Simulated VP encroachment (decreased moss dry mass and increased vascular LAI) both decreased μ* of V_cmax,moss_ and J_max,moss_. While V_cmax,moss_ still remained the most important parameter for GPP sums, J_max,moss_ became less important than V_cmax,vas_ at low m_dry_ and high LAI. This is likely caused by shading (and thus decreasing electron transport due to decreased radiation) and indicates the onset of VP dominance. Measurements of grass LAI in restored, VP encroached bogs reached values up to 5.5 m^2^ m^−2^^34^. This illustrates that LAI can be much higher than the maximum of 1.5 m^2^ m^−2^ used here, which consequently also might reduce moss coverage. The results suggest an increasing importance of VP photosynthesis in encroached bog ecosystems while the share of Sphagnum mosses will decrease. Although mosses still remained as the main contributor of GPP (at least at VP LAI up to 1.5 m^2^ m^−2^), accurately describing VP photosynthetic traits becomes more important at encroached or drained sites.

Effects of moss desiccation on GPP are prominent as all three moisture related parameters of moss photosynthesis are shown to be important, although WTD was relatively close to surface, even in summer 2018. These moisture related parameters will likely become even more important at sites with deeper WTD, like insufficiently restored sites or under strong VP encroachment. Especially Δ_desic_ showed very high importance up to comparably shallow WTD of ~ − 0.07 m below soil surface and was the third most important parameter for annual GPP. Above this threshold, its impact declined very rapidly as the capillary rise from the water table was sufficiently strong to maintain Sphagnum moisture above θ_desic_, i.e. at non-limiting levels. The strength of decline in photosynthetic capacity (Δ_desic_) seems to be of higher importance than the critical moisture value (θ_desic_). This is emphasizing the strategy of *Sphagnum* to avoid rather than to tolerate desiccation, as several studies already suggested^37^. The decreasing importance of both, Δ_desic_ and θ_desic_ at higher LAI (even more distinct for R_eco_) supports the finding of Heijmans^8^ and Nichols and Brown^9^ that grass canopy protects moss from wind and desiccation (reduced evaporation rate). Further, the role of these two parameters declined with higher m_dry_ as the increased water holding capacity reduces the amplitude of moss moisture variations. The third parameter related to hydraulic traits (θ_symp_) affects the shape of the water content response of air-chloroplast CO_2_ conductance. It showed a comparable strong relationship between μ* and WTD (Table 1). At low water levels (and consequently low moss moisture) CO_2_ diffusion is not inhibited by water films of external water. At high moss moisture θ_symp_ becomes more influential, as it describes the moisture content above which the CO_2_ conductance starts to decrease. Morris rank of θ_symp_, however, did not change linearly but showed an optimum instead, i.e. decreased rank above and below water levels close to soil surface. Consequently, other parameters become more crucial here, e.g. maximum CO_2_ conductance g_max_ (related to efficiency of air-chloroplast CO_2_ transport in non-limiting moisture) and quantum yield α (not presented due to μ* < 5 mol m^−2^). Overall, even under shallow WTD and near-natural *Sphagnum* cover as found at our test-site, moss desiccation behaviour can strongly impact ecosystem GPP and great care needs to be taken parameterizing its model representation, especially for future climate scenarios characterized by increasing magnitude and frequency of dry periods.

An effect of shading from vascular plants is visible regarding the importance of moss photosynthetic temperature response (tV_c,moss_ and tJ_moss_). Due to shading (i.e. increasing overlying vascular LAI), moss temperature becomes less variable compared to direct radiation exposure. Thus, variability of photosynthetic rates will become less dependent on temperature, and importance of temperature response parameters will decrease (Fig. 3). As moss m_dry_ per unit ground area is expected to decrease due to shading^38^ under VP encroachment (as in our synthetic experiment), the moss will hold less moisture, leading to reduced heat capacity and resistance to temperature variations (i.e. decreased cooling effect). The risks for extreme moss temperatures and desiccation seem, however, to decrease as the sheltering from direct sunlight and wind exposure reduces evaporation rates.

Modelling annual R_eco_ was strongly dependent on the soil parameters base respiration rate (r_10_) and temperature sensitivity (Q_10_). The behaviour of these parameters regarding environmental conditions was similar, and decreased in importance only at shallow water tables when the peat profile was saturated. As WTD is comparably close to surface in all cases, a reduction in microbial activity due to drought was not occurring, and sensitivity of R_eco_ to these parameters was only marginally affected by environmental conditions. Ranking of r_10_ and Q_10_ was not altered by composition of VP, indicating a high contribution of soil respiration to total R_eco_. Since dark respiration rate of both moss and VP are defined proportional to V_cmax_, the R_eco_ was sensitive to both parameters, regardless of the selection of boundaries. Thus, in contrast to GPP, moss respiration has a small and robust effect to R_eco_. Although there was a pattern of increasing importance of some VP parameters (e.g. V_cmax_ and R_d_/V_c_), they did not reach higher ranks as observed for GPP.

Overall, we demonstrated that sensitivity of CO_2_ fluxes (GPP and R_eco_) depends more on intra-annual changes of climate conditions compared to bog's plant traits or shifts in vegetation composition, i.e. there was a higher fluctuation of ranking for each parameter throughout the year than changed sensitivities of annual balances caused by wider boundaries or encroachment. This might be the result of the extreme conditions in 2018. However, despite this high variation throughout the year, parameter rankings were robust against extension of MSA boundaries. This suggests the transferability of the model's parameter importance to other temperate raised bog sites even at extreme years. We further illustrated initial dominance in photosynthesis contribution of VP traits (especially carboxylation rate) during simulated encroachment, shown by decreased rankings of moss parameters and at the same time increasing importance of VP parameters. In contrast, respiration is mainly driven by peat properties and water table regardless of vegetation composition. However, the current approach of computing R_soil_ is not distinguishing between heterotrophic and autotrophic soil respiration. Since VP roots were present in saturated conditions, respiration fostered by aerenchyma would be valuable to include in the model. Especially at increasing LAI (and consequently increasing RAI) contribution of VP to R_eco_ might be underestimated in the present results.

We conclude that pyAPES is capable for simulating CO_2_ fluxes (Fig. 6), soil temperature and water table (Fig. 5) of temperate raised bogs. Under (near-) natural conditions, carboxylation rate of moss had dominating impact on bog GPP while peat respiration was the main factor affecting R_eco_. The impact on almost all plant traits was found to be highly non-linear, i.e. very high and low parameter values led to major changes in annual CO_2_ fluxes. Thus, the extent of necessary parameter calibration highly depends on site conditions, but most important ones are robust in ranking. However, sensitivity of bog GPP and R_eco_ to plant traits can change substantially depending on climate conditions. Especially WTD (and consequently traits associated with moss moisture) showed highly fluctuating ranks and were important for annual sums even though they had very low ranks during most of the modelling period. Therefore, if possible, we recommend measurements of moss moisture as well as hydrological properties of mosses as extensive as possible due to its crucial impact on bog photosynthesis. Consequently, it is necessary to consider moisture related traits even at comparable shallow WTD in raised bogs. During encroachment, VP related parameters became increasingly important. Most important upcoming traits were carboxylation and electron transport rate. This gives evidence that even at only slightly encroached bog sites photosynthetic traits of vascular start to become relevant for CO_2_ balance. Here, moss V_cmax_ still remained the most important trait but decreased in absolute importance (μ*) inversely to VP. We were able to delineate the most relevant plant physiological traits determining ecosystem carbon fluxes dependent on climate conditions and vegetation composition, which is useful for future modelling CO_2_ fluxes from raised bog sites.

## Methods

### Site description

**Figure 4:**
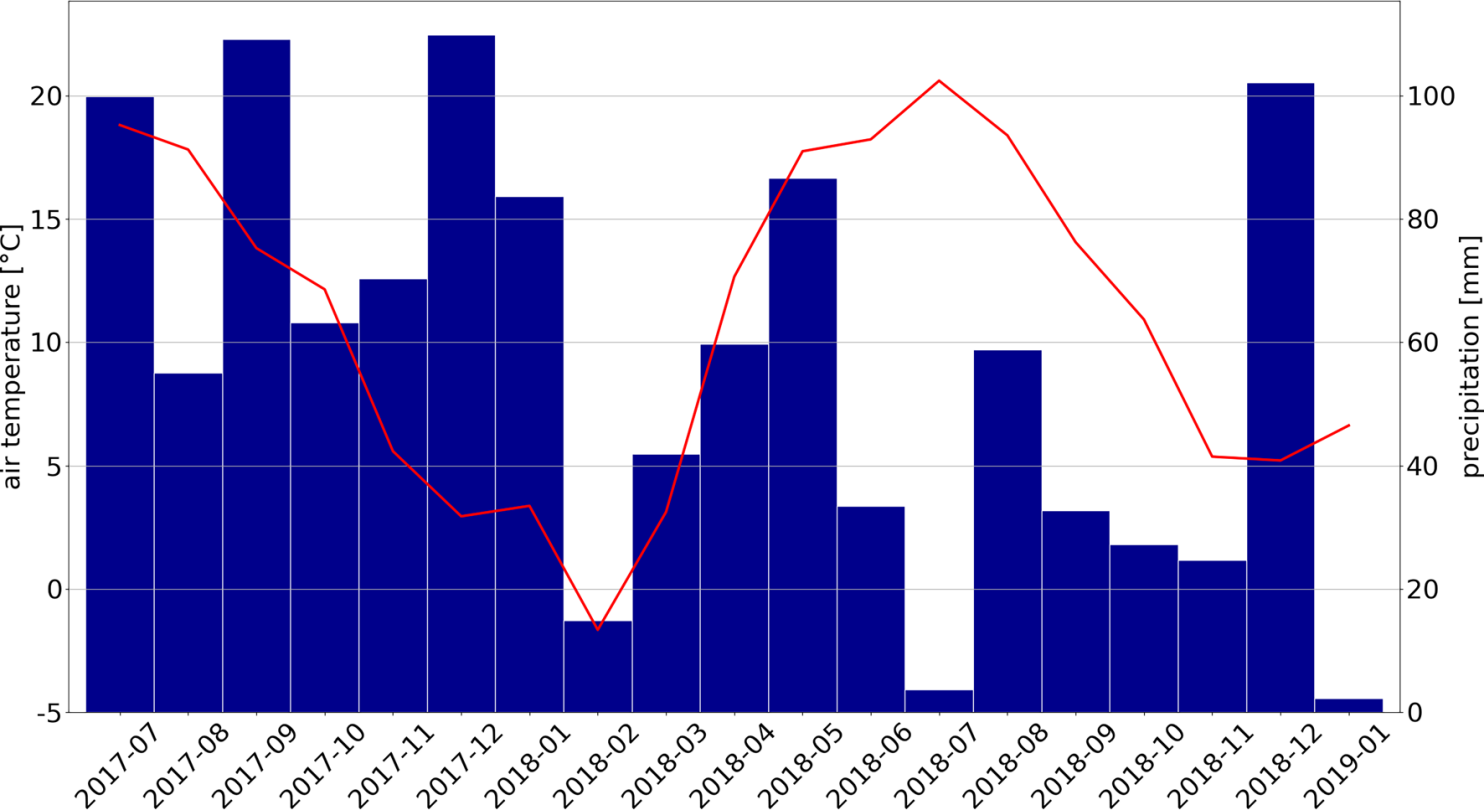
Climatic conditions at Meerkolk site during modelling period. Figure shows monthly means of air temperature (line) and monthly sums of precipitation (bars). Over the whole 1.5-year modelling period average temperature was 11.1 °C and sum of precipitation was 1079 mm (719 mm yr^-1^).

The model parameterization and sensitivity analysis were conducted using 'Meerkolk' bog, a nature conservation area located in Emsland, Northern Germany (52°38′N; 07°08′E) as a test area. With its peat thickness (3.5 m), vegetation cover (100% total coverage; 100% *Sphagnum*; 15% VP), water table depth WTD (− 0.02 m annual average) and peat properties, it owns characteristics of near-natural raised bogs in temperate climate conditions. The underlying peat was classified as Ombric Fibric Histosol^39^ with an increasing degree of peat decomposition with depth from Von Post degrees^40^ of H1 to H3 within the upper three horizons (53 cm depth) and H6 to H9 below. Mean annual precipitation is 791 mm and mean annual temperature is 9.8 °C according to a weather station of the German Weather service in Lingen (nearby Meerkolk site), documented from 1971 to 2000. During the modelling period (2017-07-01 to 2019-01-01) the sum of precipitation, aggregated to annual scale was 719 mm year^−1^ and average temperature was 11.1 °C. Monthly average temperatures and sums of precipitation are shown in Fig. 4. Most abundant VP species were *Rhynchospora alba*, *Erica tetralix*, *Vaccinium oxycoccos* and *Eriophorum angustifolium*. For a more detailed site description see Oestmann et al.^22^.

VP one-sided leaf area index (LAI m^2^ m^−2^) was determined at the end of summer 2018 using LI-COR LAI-2200C Plant Canopy Analyzer. Measurements were replicated at three plots to capture spatial heterogeneity and conducted inside the frames of the flux measurement chambers (75 × 75 cm). At each plot eight above and eight below measurements were conducted, using a 45° angle view cap with view direction to the centre, as suggested in the LI-COR LAI 2200c manual for point measurements. Results were computed with the LI-COR 'FV2200' software (version 2.2.1). All measurements were averaged to yield a representative mean of annual maximum LAI of 0.47 m^2^ m^−2^.

Dominating bryophyte species was *Sphagnum papillosum.* Moss dry biomass m_dry_ [kg m^−2^] was determined using a grid of 5 × 5 cm squares covering the whole plot resulting in overall 225 sections. Number of moss capitula were counted in every second section and scaled to square meter. Ten moss shoots (only green, i.e. photosynthetically active parts) per plot were sampled and length was measured, resulting in an overall mean of 3.5 cm. We assume that moss length is equal to moss height h_m_. Subsequently, samples were oven dried at 60 °C for at least 48 h to determine dry biomass m_dry_ [kg m^−2^]. Moss dry mass was then scaled-up using capitula counts of all sections:

1 $${m}_{dry,i}=\frac{1}{113}*\sum_{j=1}^{113}{(N}_{j}*{\overline{m} }_{s,i}*{a}_{j}^{-1})$$

where ${\overline{m}}_{s}$ [kg shoot^−1^] is the average dry mass of the sample, N represents the quantity of moss capitula, *a* defines the section area [m^−2^] and i and j are indices for plots and sections, respectively.

m_dry_ is the average of the three plots (0.27 kg m^−2^). Living moss bulk density (ρ_m_) is the result of m_dry_ divided by moss height (h_m_) and equals 7.48 kg m^−3^.

Further, we measured maximum water content of moss layer w_max_ that can be retained against gravity. Ten sample cylinders with 3.5 cm height were cut from the moss carpet and saturated with water. Subsequently, samples were drained on suction plates applying a suction of 0 cm at the bottom of the cylinders until weight constancy. Mass was measured for drained and oven dried (105 °C) samples and w_max_ was determined from the mean difference of both. h_m_, ρ_m_ and w_max_ were used for model parametrization.

All measurements are summarized in Table 2.

**Table 2:** Measured plant properties at Meerkolk site.

Parameter	Description	Unit	Value
h_m_	Moss height	m	0.036
m_dry_	Moss dry mass	kg m^−2^	0.27
ρ_m_	Moss bulk density	kg m^−3^	7.48
w_max_	Maximum moss layer water content	g g^−1^ m_dry_	34.0
LAI_max_ vas	Maximum leaf area index of VP	m^2^ m^−2^	0.47
LAI_min_ vas	Minimum leaf area index of VP	Relative	0.21

### Calibration data

For Meerkolk site, continuous measurements of soil temperature (T_s_) and water table depth (WTD) at 30 min resolution, and campaign-wise data on gross primary production (GPP) and ecosystem respiration (R_eco_) were available. In the following, negative carbon fluxes indicate uptake, while positives indicate release of carbon. T_s_ at 2 cm soil depth were measured by a weather station at a site close to Meerkolk with similar site conditions. WTD [m] was determined using groundwater pressure transducers; negative signs indicate WTD is below soil surface.

In total 18 measurement campaigns were conducted to measure GPP and R_eco_ at each of the three replication plots. While NEE was determined with transparent chambers, measurements of R_eco_ were conducted using opaque chambers. The measurements covering the whole amplitude of photosynthetic active radiation (PAR) and T_s_, starting an hour before sunrise (lowest values) and reaching maximum values in the afternoon. GPP was calculated by subtracting R_eco_ measurements from the nearest in time NEE measurement. The measurements and data-processing is presented in detail in Oestmann et al.^22^.

### Implementation of photosynthesis in pyAPES

The pyAPES is a multi-layer, multi-species soil–vegetation–atmosphere transfer model, which models ecosystem GPP, R_eco_, evapotranspiration, sensible heat and momentum fluxes using 1-D representation of the canopy-moss-soil continuum. Both moss and VP photosynthesis are implemented using Farquhar-von Caemmerer-Berry (FvCB) biochemical model^23^, the standard approach for modelling CO_2_ assimilation in vascular plants^41^ and mosses^42–44^. The solution of leaf (or moss layer) photosynthesis is coupled with solution of energy balance (leaf/moss temperature and (stomatal) conductance), using iterative solutions between the leaf/moss processes and the ambient microclimatic gradients (CO_2_, air humidity and temperature) and short and long-wave radiation regime. For complete model description see Launiainen et al.^20^ and for applications in boreal sites^21,45,46^. The model assumes, that net assimilation rate (A_n_) is either limited by Rubisco (A_c_ rate) or regeneration of Ribulose-1,5-bisphosphate (A_j_ rate):

2 $${A}_{n}=min\left\{{A}_{c},{A}_{j}\right\}-{R}_{d}$$

where Rd is the dark respiration rate. For the subsequent sensitivity analysis, we used maximum carboxylation velocity (V_cmax_), defining A_c_, as well as maximum transport rate of electrons (J_max_) and quantum yield (α) which determine A_j_. The V_cmax_, J_max_ and R_d_ temperature response follows Medlyn et al.^24^, and we included optimum temperature of V_cmax_ and J_max_ and activation energy of R_d_ into the sensitivity analysis.

Optimal stomatal conductance of VP (g_s_^*^) is described in pyAPES following the Unified Stomatal Optimization (USO) approach of Medlyn et al.^47^:

3 $${g}_{s}^{*}\approx {g}_{0}+\left(1+\frac{{g}_{1}}{\sqrt{VPD}}\right)\frac{A}{{C}_{a}}$$

where g_0_ is residual conductance, g_1_ stomatal slope, C_a_ the ambient CO_2_ concentration, A the net assimilation rate [Eq. (2)] and VPD the water vapour deficit (VPD). The g_0_ and g_1_ were also included in the MSA.

For mosses, CO_2_ conductance decreases exponentially with water content (θ), based on an empirical function fitted to data of Williams and Flanagan^44^ for *Sphagnum* and *Pleurozium*:

4 $$g={g}_{max}*\left({a}_{0}*{e}^{{a}_{1}*\left(\theta -{\theta }_{symp}\right)}+1.0-{a}_{0}\right)$$

With g_max_ describing CO_2_ conductance at maximum symplast water content (θ_symp_), i.e. when all external water is evaporated and a_0_ and a_1_ are empirical parameters of the fitted function. g_max_ is thus actually a shape parameter instead of maximum conductance.

The effect of desiccation on photosynthetic capacity (cap_photo_) at low moss moisture content (a multiplier for moss V_cmax_) is described as

5 $$ca{p}_{photo}=0\le 1.0+{\Delta }_{desic}*log\left(\frac{\theta }{{\theta }_{desic}}\right)\le 1$$

Thus, desiccation leads to decrease of moss assimilation capacity (V_cmax_, J_max_) and dark respiration rate (R_d_) if moss water content (θ) is lower than a threshold (θ_desic_), while the strength of the decay is defined by the slope parameter Δ_desic_. Both reduction and recovery of photosynthesis is assumed instantaneous and reversible in the model. Both Δ_desic_ and θ_desic_ were included in MSA. The moss moisture content is modelled at 30 min timestep, and accounts for rainfall interception, evaporation as well as capillary rise from underlying soil (affected by WTD and peat hydraulic properties).

### Model parametrization and adaptation to site conditions

Measurements of T_s_, WTD and carbon dioxide fluxes, i.e. GPP, R_eco_ and NEE were used to calibrate the model for temperate bog. Wherever possible we used measured values for parametrization. If no measurements were available, we conducted a literature review. In contrast to VP, literature values of *Sphagnum* moss hydraulic or photosynthetic properties are rather scarce. When no value could be found for a certain parameter, we used values from VP studies instead. If this was still not possible, we inspected respective functions and set parameter values to best fit measurements and plausibility of related model outputs.

In a first step we calibrated the heat flow of pyAPES' soil sub model using measured T_s_ in 2 cm soil depth. No adaptation of parameters was necessary here, since observations were overall well reproduced by pyAPES with a RMSE of 2.5 °C. However, in some periods (mainly summer 2018), modelled T_s_ is oscillating stronger than measurements.

**Figure 5:**
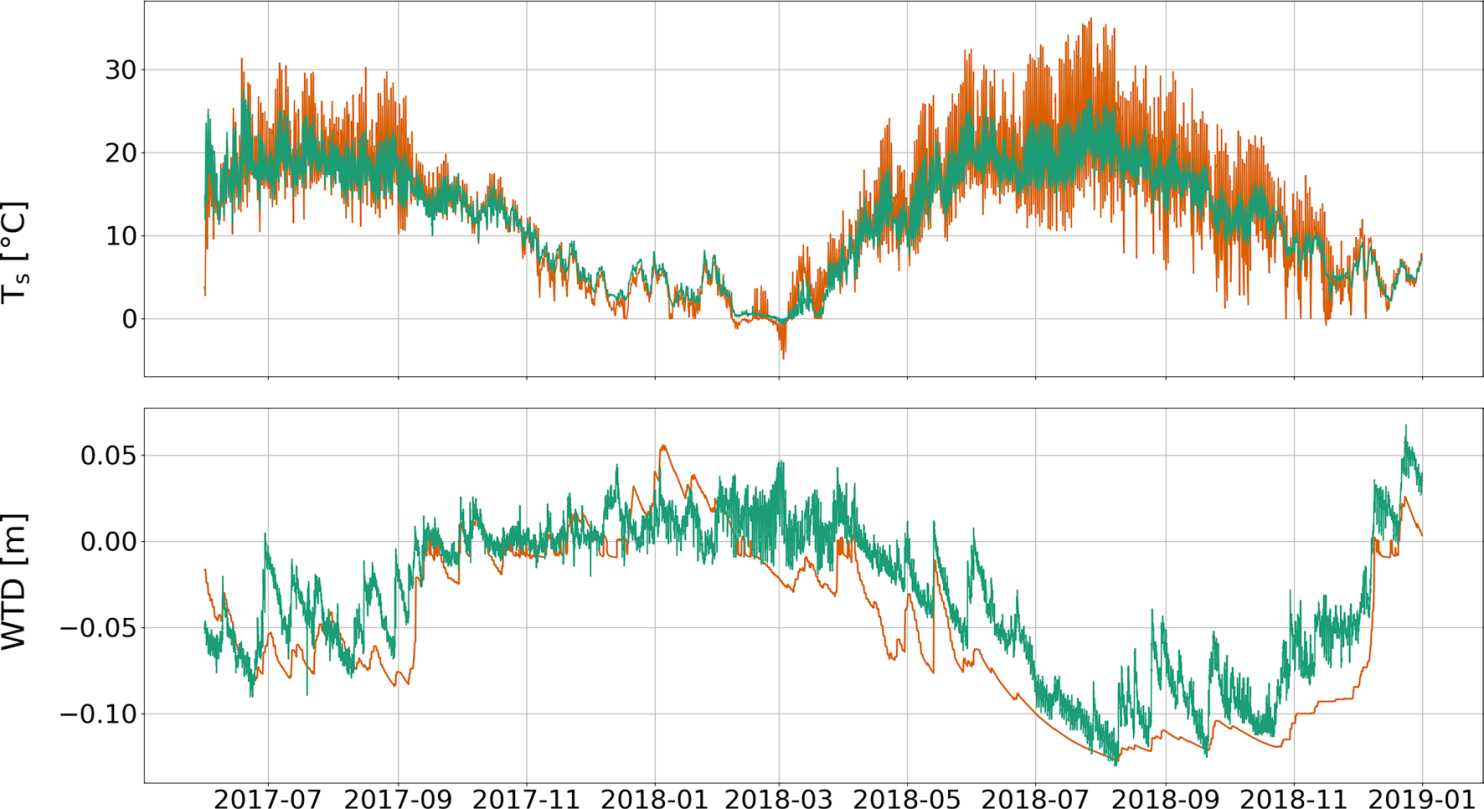
Measured (green) and modelled (orange) soil temperatures at 2 cm depth (T_s_, top, RSME = 2.5 °C) and water table depth (WTD, bottom, RSME = 2.8 cm). A negative sign of WTD indicate water table is below surface.

As several studies have pointed out the importance of moss moisture for photosynthetic capacity^37,48^, we decided to calibrate WTD subsequently. It acts as a proxy for moss moisture, from which no data was available. Simulated moss moisture and capillary rise were observed carefully to show realistic dynamics. Calibrating the model to water table depth was challenging, and parameters of the water retention curve (WRC)^49^ of both living moss and peat were most crucial for this part. We tested numerous sets of retention parameters for *Sphagnum* peat reported in McCarter and Price^25^, but none of these could reproduce WTD dynamics during the hot and dry summer of 2018. Finally, we applied a mixture of Liu & Lennartz^27^ for the air entry value α, and Price et al.^26^ for the shape parameter n to the upper three soil horizons. Hooghoudt equation^50^ describes lateral flow in the model. At Meerkolk, lateral drainage to the surrounding, lower agricultural areas plays a significant role, especially in times of water excess. Thus, amount and depth of lateral outflow was set to well represent winter water tables. Finally, the model was able to reasonably well reproduce the dynamics of changing water tables, even in summer 2018, with a RMSE of 2.8 cm (Fig. 5).

Water retention parameters of living moss micropores are, similar to the peat soil horizons, described by Van Genuchten Mualem equation, following the analogy of Voortman et al.^51^. The authors summarize several studies, showing high variations for α and n, as well as differences in their combination to define the shape of water retention curve. In the model the water content at saturation (θ_sat_) and residual water content (θ_res_) are defined by maximum gravimetric water (w_max_) content of moss and maximum symplast water content (θ_symp_), respectively, which is implemented in pyAPES as a ratio of w_max_. They are converted in the model to volumetric water contents using moss bulk density (ρ_m_).

6 $${\theta }_{sat}=\frac{{w}_{max}}{{\rho }_{w}}*{\rho }_{m}$$

7 $${\theta }_{res}=\frac{{\theta }_{symp}}{{\rho }_{w}}*{\rho }_{m}$$

With w_max_ = maximum moss water content [g g^−1^ dry weight], θ_symp_ = maximum symplast water content [g g^−1^ dw], ρ_w_ = water density [g m^−3^] and ρ_m_ = moss bulk density [g m^−3^].

Using measured ρ_m_, moss WRC was very narrow and default α and n parameters resulted in a too strong decrease in WTD in dry periods. We increased the n value, which improved the modelled WTD but caused a break in capillary rise in summer 2018. By enhancing minimum hydraulic conductivity this issue could be solved. Roughness height of moss was further set to 1/10 of h_m_.

As VP are not abundant in Meerkolk, maximum vascular LAI is 0.47 m^2^ m^−2^ on average. This value only represents a maximum LAI, which is not sufficient for model parametrization. To display seasonal dynamics, measurements from a comparable site^34^ were used to calibrate development of modelled LAI by adjusting the base temperature for degree-day calculation for phenological development simulation (Fig. S1).

Hájek^52^ provides an overview about optimal moisture of *Sphagnum papillosum* (dominating moss species in Meerkolk) for photosynthesis. θ_desic_, Δ_desic_ and θ_symp_ were set to best fit values presented in Hájek^52^, supported by Schipperges and Rydin^48^ (Fig. S2).

In the default settings of pyAPES, Farquhar parameters (V_cmax_, J_max_ and R_d_) for moss community are presented per unit ground area, and light attenuation within the single-layer moss is not accounted for. Since one of our research questions aims at changing vegetation composition (and thus changing m_dry_), it was necessary to include a scaling of Farquhar parameters with moss dry mass. Currently, published relationships of V_cmax_ etc. and moss dry mass are scarce, however at least J_max_ seems to decrease almost linearly up to a depth of ca. 4 cm in a *Pleurozium schreberi* moss canopy^46^ and non-linear functions with respective shape parameters are missing in the literature, we decided for a simple linear scaling with m_dry_.

Maximum velocity of carboxylation (V_cmax_) for VP was set to average values of Ekberg et al.^53^ for *Eriophorum angustifolium* at 20 °C. Maximum electron transport rate (J_max_) and basic dark respiration rate (R_d_) are set as ratios of 1.97^48^ and 0.03, respectively of V_cmax_, as for mosses.

Heterotrophic and autotrophic soil respiration (R_soil_) in pyAPES is computed using the approach of Pumpanen et al.^54^ and moisture response of Skopp et al.^55^. By default, R_soil_ is scaled by relative root area density in the respective soil layer. For bogs with shallow water table, we assume bulk peat respiration takes place only in the unsaturated zone. R_soil_ is thus computed as layer-thickness weighted average from all soil layers above WTD, assuming no respiration within water saturated layers:

8 $${R}_{soil}=\sum_{i=1}^{n}{(R}_{i}* \frac{{dz}_{i}}{{z}_{n}})$$

where R and dz are the soil respiration and thickness of layer i, z the soil depth and the subscript n denotes the index of the corresponding layer of current WTD.

**Figure 6:**
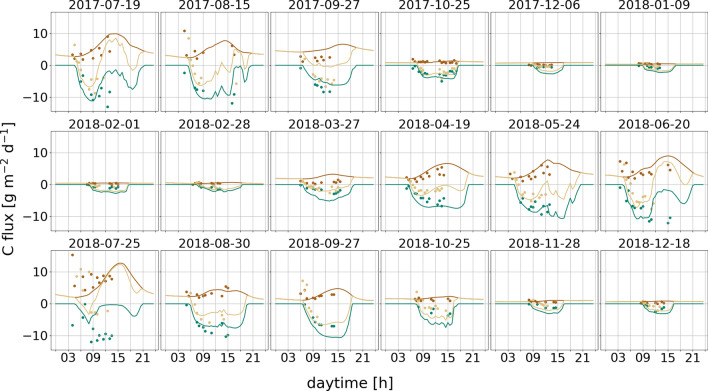
Measured (dots) and simulated (lines) fluxes of GPP (green), R_eco_ (brown) and NEE (beige) for each measurement campaign during the modelling period.

Using the parametrization of Table S3, the model slightly overestimated fluxes. Due to higher overall GPP bias (− 0.7 g CO_2_ m^−2^ day^−1^) compared to R_eco_ (0.5 g CO_2_ m^−2^ day^−1^), simulated NEE is tending to show more CO_2_ uptake (− 0.6 g CO_2_ m^−2^ day^−1^) than observed (more details in Table S2). Both, bias and RMSE varied strongly between campaigns and had lowest errors in winter months (at low fluxes) and highest in summer (Fig. 6). All adapted parameters of pyAPES are presented in Table S3.

### Morris sensitivity analysis

To find most relevant parameters of the pyAPES’ carbon module for bog GPP and Reco, sensitivity analysis was conducted using the approach of Morris (MSA)^56^. The method is based on evaluation of elementary effects (EE), i.e. evaluating the alteration of output variables by adjusting only one factor at a time (OAT). These local OAT approaches are less expensive in computation time, as they need less model runs in contrast to global approaches. However, different from other OAT methods, Morris approach uses randomly sampled parameter combinations. Thus, the whole input space of parameters is covered and Morris method can be seen as computationally less expensive approximation of global sensitivity analysis.

To compute an EE of one parameter, a base parameter set must be created. For this purpose, lower and upper boundaries must be defined for each of the *k* parameters. Within these boundaries, a parameter can take a value out of *p* levels in an even distance from each other. This results in a *k* times *p* dimensional grid, representing the input space and called Ω. From Ω a random value is drawn for each parameter *X*_i_ to get the base parameter set. In a next step, each X_i_ is changed by Δ, without returning to its initial base value. This procedure requires k + 1 parameter sets (base set plus k changes, one for each X_i_) and is called a trajectory. One trajectory yields exactly one EE for each input parameter. Repeating this procedure *r* times, one receives a distribution of EE, F_i,_ for each input parameter with computational costs of *r*(*k* + 1) model evaluations. Mean value μ, standard deviation σ can now be computed from F_i_. According to the suggestions of Campolongo et al.^57^, a distribution of absolute values from EE, G_i_ (and whose mean value is called μ*), is used in this study, to avoid nullifying effects of opposite signs when the model is non-monotonic. The coefficient of variation (σ/μ*) can give evidence whether the effect of an input parameter is (i) linear and additive, (ii) non-linear or/and interacting or (iii) negligible.

In this study, parameters were allowed to vary at four levels within given boundaries. For each distribution G_i_, 1000 bootstrap runs were conducted to calculate a 95% confidence interval (CI) for μ*. Following the approach of Campolongo et al.^57^, 1000 trajectories were created and 50 optimal were chosen for the analysis. 'Optimal' in this context means reaching the widest spread over the whole input space (Euclidean distance). As the number of trajectories for original Morris analysis is between 10 and 50^51^, we used 50 optimal trajectories for the enhanced method. To evaluate whether this number of optimal trajectories leads to reliable results we tested different seeds (i.e. starting points for generating pseudo-random numbers) and compared rankings of the respective analysis. Ranking of the parameters was the same for all seeds, considering their confidence intervals, when using 50 optimal trajectories. Final analysis was conducted without setting any seed. Parameter were ranked according to their amount of μ* without considering their confidence intervals. Parameters with equal μ* all got the lowest ranking of this group (e.g. [1, 2, 5, 5, 5, 6, …]).

Overall, 22 parameters were investigated (Table S3). Kattge and Knorr^58^ reported a linear relationship between J_max_ and V_cmax_ at 25 °C, but found this ratio to be depended on growth temperature. Thus, we decided to define both, J_max_ and R_d_ as ratios of V_cmax_ and varied V_cmax_ and these ratios within MSA.

We first investigated sensitivity of annual balances while varying parameters within standardized boundaries as well as broader boundaries (i.e. literature observations). Further we analysed the impact of changing site conditions and vegetation composition to flux sensitivities. For all computations regarding Morris sensitivity analysis Pythons SALib package version 1.4.5^59^ was used.

### Sensitivity of annual balances

To identify the most crucial parameters for modelling of the overall bog CO_2_ exchange, annual balances of GPP and R_eco_ were used as output variables in the SA. Parameter boundaries were defined to be ± 30% of the parametrization used to reproduce best the observations of the study site. Using a uniform boundary definition among all parameters minimized impacts on SA results and thus enabled a standardized ranking. In a second step, boundaries were expanded to the broader range of measured parameter values found in a literature review, to investigate whether parameter ranking is likely to change when applying the model to other bog sites. Analogous to model parametrization we derived values from VP if literature did not provide moss parameters.

J_max_/V_cmax_ ratios are either reported directly or calculated from both values. Same ranges for θ_desic_ and θ_symp_ were used, as most studies report optimal water contents for photosynthesis but not distinguish between desiccation and CO_2_ diffusion.

### Response of parameter sensitivities to seasonal site condition dynamics

To identify, whether environmental conditions such as WTD or T_s_ affected carbon flux sensitivities, Morris analysis was conducted not only for annual sums, but also for each timestep in half-hourly resolution over the simulation period. Sensitivities of carbon fluxes with respect to concurrent simulated WTD and T_s_ were evaluated to assess how the parameter ranking is affected by seasonal variability in the environmental conditions (research question II).

Thus, average elementary effects could be computed for each timestep. As WTD and T_s_ values were possibly affected by changing parameters, WTD and T_s_ timeseries were sampled from each individual model run within the SA. Following this approach for all trajectories, mean values of WTD and T_s_ distributions for all timesteps were computed. We investigated the five most influential parameters from SA with standardized boundaries. Linear regression analysis was conducted using R software (RStudio version 1.3.959) to find significant relationships. A regression model of the form Y = a + b × WTD + c × T_s_ was used.

### Response of flux sensitivities to changing composition of plant functional groups

In a case of VP encroachment, increase of VP LAI and decrease of moss biomass is expected due to shading^38^. To investigate the impact of vegetation composition on flux sensitivities and parameter importance, we generated synthetic parameter sets in the range of 0.5–1.5 m^2^ m^−2^ LAI_max_ and 0.15–0.35 kg m^−2^ m_dry_. To set the upper and lower boundary for LAI_max_ and m_dry_, respectively, we used measured values from another bog site in Northern Germany with strong encroaching vascular species. The vegetation composition of the Meerkolk experimental site well represents near-natural bog vegetation composition. Observed LAI_max_ and m_dry_ ranges were divided in five even spaced steps and Morris analysis was conducted once for each combination within this 5 × 5 matrix.

### Supplementary Information

Supplementary Information.

## Data Availability

Data and model code will be made available on request from the authors.
