# Supplementary material for: Impact of vegetation composition and seasonality on sensitivity of modelled CO2 exchange in temperate raised bogs
Source: Sci Rep. 2024 May 14;14:11023. doi: 10.1038/s41598-024-61229-6 (PMC11094101; doi:10.1038/s41598-024-61229-6)
Supplement: Supplementary file 1 — Supplementary Information. [file 41598_2024_61229_MOESM1_ESM.docx]

# Supplementary Information

Supplementary Table S1: List of abbreviations.

| **Abbreviation** | **Explanation** | **Unit** |
| --- | --- | --- |
| α (vGM model) | air entry value | cm^-1^ |
| α (Farqhuar model) | quantum yield | mol mol^-1^ |
| Δ_desic_ | slope of photosynthetic decrease at θ < θ_desic_ | - |
| g_max_ | moss CO_2_ conductance at θ_symp_ (shape parameter) | mol m^-2^ s^-1^ |
| g_0_ | residual stomatal CO_2_ conductance | mol m^-2^ s^-1^ |
| g_1_ | stomatal slope | kPa^0.5^ |
| h_m_ | moss height | m |
| J_max_ | maximum transport rate of electrons | µmol m^-2^ s^-1^ |
| J/V_c_ | ratio between J_max_ and V_cmax_ | - |
| K_unsat_ | minimum unsaturated conductivity | m s^-1^ |
| LAI | leaf area index of VP | m^2^ m^-2^ |
| m_dry_ | moss dry mass | kg m^-2^ |
| MSA | Morris sensitivity analysis | - |
| Q_10_ | temperature response of r_10_ | - |
| ρ_m_ | moss bulk density | kg m^-3^ |
| r_10_ | base soil respiration at 10 °C | μmol m^-2^ s^-1^ |
| R_d_ | dark respiration rate | µmol m^-2^ s^-1^ |
| R_d_/V_c_ | ratio between R_d_ and V_cmax_ | - |
| tJ | temperature optimum of J_max_ | °C |
| tR_d_ | activation energy of dark respiration (parameter of temperature response) | kJ mol^-1^ |
| tV_c_ | temperature optimum of V_cmax_ | °C |
| T_s_ | soil temperature | °C |
| θ_sat_ | saturated water content (vGM parameter) | m^3^ m^-3^ |
| θ_res_ | residual water content (vGM parameter) | m^3^ m^-3^ |
| θ_desic_ | water content where photosynthetic capacity starts to decrease | g g^-1^ |
| θ_symp_ | maximum symplast water content | g g^-1^ |
| V_cmax_ | maximum velocity of carboxylation | µmol m^-2^ s^-1^ |
| vGM | van Genuchten-Mualem model | - |
| VP | vascular plant(s)^*^ | - |
| w_max_ | maximum moss water content | g g^-1^ |
| WTD | water table depth | m |

* for Meerkolk only grass species


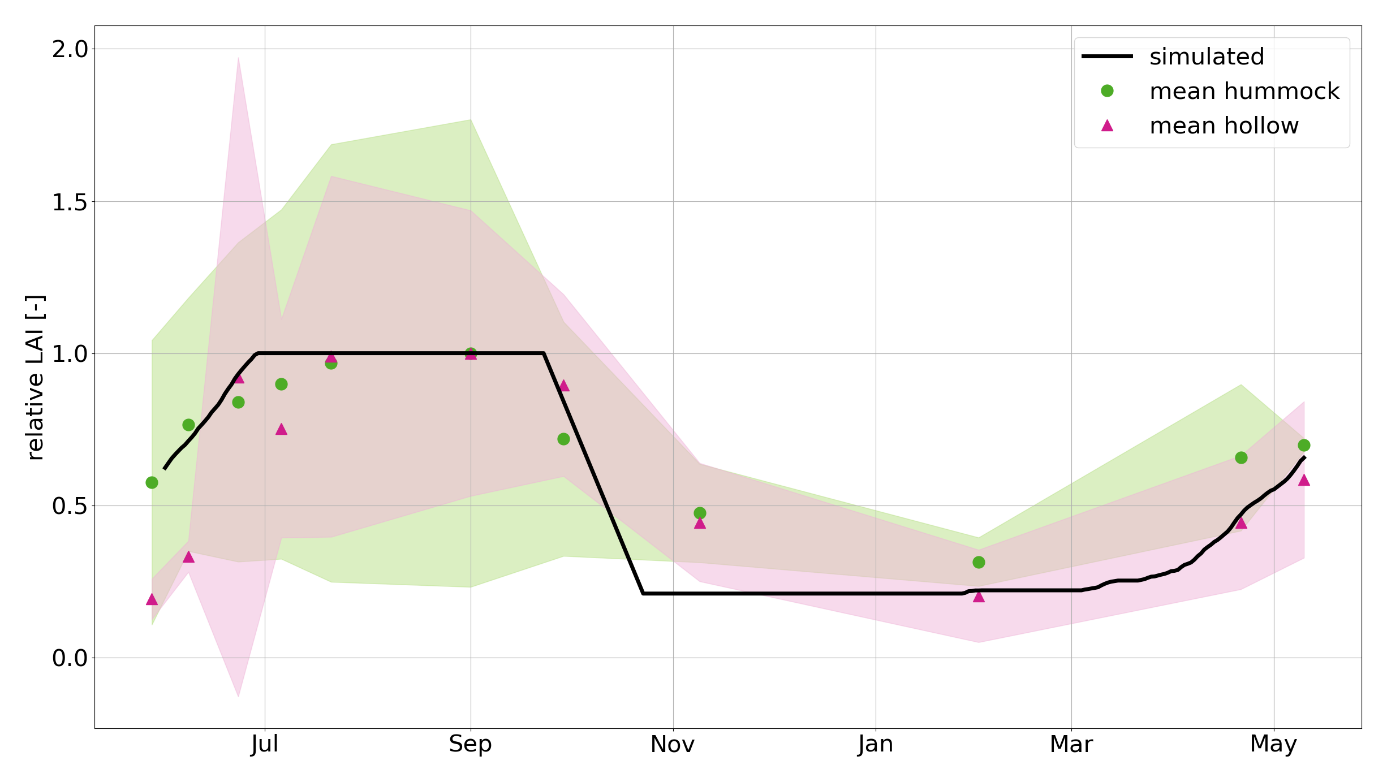


Supplementary Figure S1: Relative seasonal development of modelled (black line) and measured (hummocks: green dots, hollows: purple triangles) VP leaf area index (LAI). Equivalent to Meerkolk site the measured values for hummock and hollow represents mean values of each three replicates and shaded area their respective standard deviation. Values are standardized to its respective maximum value in order to provide better comparison of phenological dynamics.


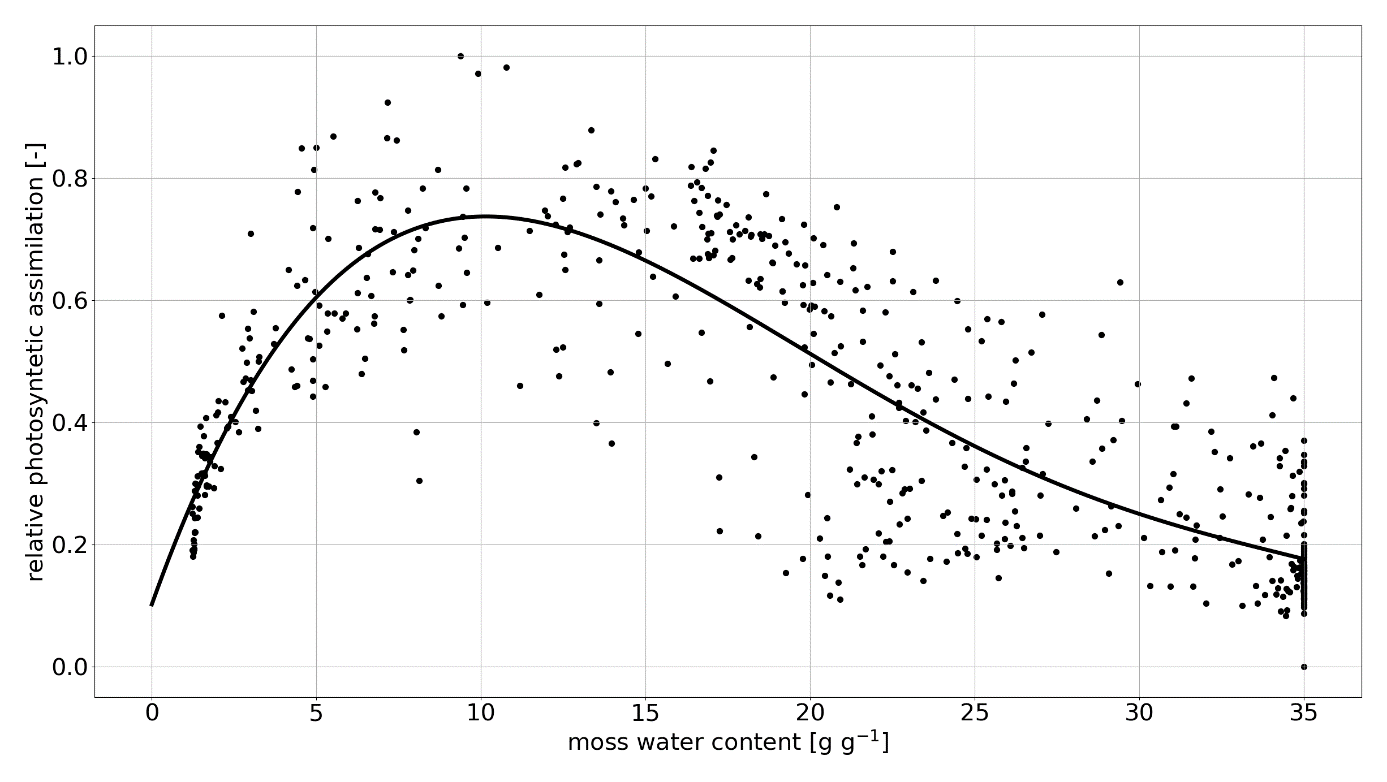


Supplementary Figure S2: Relationship between modelled photosynthetic capacity relative to its maximum value and daily mean of gravimetric moss water content.

Supplementary Table S2: Root mean squared errors and bias of simulated vs measured data for GPP, R_eco_ and NEE of pooled data of all measurement campaigns (overall) as well as minimum and maximum values. Units are [g CO_2_ m^-2^ d­^-1^].

|  | **RMSE** | | | **Bias** | | |
| --- | --- | --- | --- | --- | --- | --- |
|  | Overall | Min campaign | Max campaign | Overall | Min campaign | Max campaign |
| **GPP** | 2.4 | 0.8 | 4.4 | -0.7 | -3.1 | 3.1 |
| **R_eco_** | 2.1 | 0.2 | 4.9 | 0.5 | -2.5 | 2.5 |
| **NEE** | 2.9 | 0.4 | 6.9 | -0.6 | -4.0 | 4.2 |

Supplementary Table S3: Model parameters and their standardized and literature-based boundaries of parameters included in Morris sensitivity analysis (MSA). Parameters denoted with '-' were not part of MSA.

| **Parameter** | **unit** | **Value for adapted parameterization** | **Reference(s) for parameter value*** | **Standardized boundaries** | **Literature boundaries** | **Reference(s) for MSA boundaries** | **comments on MSA boundaries** |
| --- | --- | --- | --- | --- | --- | --- | --- |
| *Sphagnum* | | | | | | | |
| Roughness height | m | 0.003 | 10 % of h_m_ | - | - | - | - |
| n | - | 2.8 | Adjusted to fit measurements | - | - | - | - |
| V_cmax_ | μmol m^-2^ s^-1^ | 45.0 | default | 31.5 – 58.5 | 7.1 – 76.3 | Carriquí et al.^60^ | Different moss species but *Sphagnum* not included |
| J/V_c_ | - | 1.97 | Kattge & Knorr^58^ | 1.38 – 2.56 | 0.98 – 1.78 | Carriquí et al.^60^ | Different moss species but *Sphagnum* not included |
| R_d_/V_c_ | - | 0.03 | default | 0.021 – 0.039 | 0.003 – 0.05 | Carriquí et al.^60^ | Different moss species but *Sphagnum* not included |
| α (quantum yield) | mol mol^-1^ | 0.3 | default | 0.21 – 0.39 | 0.24 – 0.51 | Laine et al.^61^ | Different *Sphagnum* species at different photon flux densities |
| g_max_ | mol CO_2_ m^-2^ s^-1^ | 40.0 | default | 28.0 – 52.0 | 1.36 – 89.63 | Carriquí et al.^60^ | Different moss species but *Sphagnum* not included |
| tV_c_ | °C | 40.0 | Adjusted to fit measurements | 28.0 – 52.0 | 27.6 – 53.3 | Medlyn et al.^23^,  Kattge & Knorr^58^ | Different VP species |
| t_J_ | °C | 40.0 | Adjusted to fit measurements | 28.0 – 52.0 | 19.5 – 38.7 | Medlyn et al.^23^,  Kattge & Knorr^58^ | Different VP species |
| tR_d_ | kJ mol^-1^ | 33.0 | default | 23.1 – 42.9 | 53.0 | Lloyd et al.^62^ | *Macadamia integrifolia* and *Litchi chinensis* trees; also used for *Sphagnum* and *Pleurozium* in Williams & Flanagan^44^ |
| θ_desic_ | g g^-1^ | 10.0 | adjusted to fit moisture optimum reported in Hájek^37^ | 7.0 – 13.0 | 6.0 – 30.0 | Hájek^37^ | Range of optimal water contents for photosynthesis of different *Sphagnum* species |
|  |  |  |  |  | 6.3 (18% of w_max_) | Hájek & Beckett^63^ | *S. fuscum*  photosynthetic activity started to decline at ~18% of shoot water content |
| Δ_desic_ | - | 0.3 | adjusted to fit moisture optimum reported in Hájek^37^ | 0.21 – 0.39 | 0.1 – 0.6 | - | Arbitrary extension due to missing references |
| θ_symp_ | g g^-1^ | 15.0 | adjusted to fit moisture optimum reported in Hájek^37^ | 10.5 – 19.5 | 6.0 – 30.0 | Hájek^37^ | Range of optimal water contents for photosynthesis of different *Sphagnum* species |
| K_unsat_ | m s-1 | 10^-9^ | Adjusted to fit measurements | - | - | - | - |
| vascular plants | | | | | | | |
| V_cmax_ | μmol m^-2^ s^-1^ | 48.75 | Ekberg et al.^53^ | 34.13 – 63.38 | 53.5 – 76.5 | Schedlbauer et al.^64^ | *Eriophorum vaginatum* |
| J_max_/V_cmax_ ratio | - | 1.97 | Kattge & Knorr^58^ | 1.38 – 2.56 | 1.05 – 2.72 | Kattge & Knorr^58^ | Different VP species |
| R_d_/V_cmax_ ratio | - | 0.023 | default | 0.016 – 0.030 | 0.01 – 0.023 | Ekberg et al.^53^ | *Eriophorum angustifolium* |
| α (quantum yield) | mol mol^-1^ | 0.2 | default | 0.14 – 0.26 | 0.3 – 0.8 | Janka et al.^65^ | *Dendranthema grandiflora* at different irradiance and temperature levels |
| g_0_ | mol m^-2^ s^-1^ | 0.005 | default | 0.0035 – 0.0065 | -0.044 – 0.033 | Medlyn et al.^47^ | Different tree species |
| g_1_ | kPa^0.5^ | 2.1 | default | 1.47 – 2.73 | 1.66 – 12.13 | Medlyn et al.^47^ | Different tree species |
| tV_c_ | °C | 40.0 | Adjusted to fit measurements | 28.0 – 52.0 | 27.6 – 53.3 | Medlyn et al.^24^,  Kattge & Knorr^58^ | Different VP species |
| tJ | °C | 40.0 | Adjusted to fit measurements | 28.0 – 52.0 | 19.5 – 38.7 | Medlyn et al.^24^,  Kattge & Knorr^58^ | Different VP species |
| tR_d_ | kJ mol^-1^ | 33.0 | default | 23.1 – 42.9 | 53.0 | Lloyd et al.^62^ | *Macadamia integrifolia* and *Litchi chinensis* trees |
| Base temperature | °C | 2.0 | Adjusted to fit measurements | - | - | - | - |
| Σ DD maturity | °C | 1000 | Adjusted to fit measurements | - | - | - | - |
| peat soil | | | | | | | |
| α (first horizon) | cm^-1^ | 0.8 | Lui & Lennartz^27^ | - | - | - | - |
| n (first horizon) | - | 2.5 | Price et al.^26^ | - | - | - | - |
| drainage depth | m | 0.01 | Adjusted to fit measurements | - | - | - | - |
| Drain spacing | m | 1.0 | Adjusted to fit measurements | - | - | - | - |
| Maximal pond storage | m | 0.1 | measured | - | - | - | - |
| r_10_ |  | 2.5 | default | 1.75 – 3.25 | 1.2 – 3.0 | Shi et al.^66^ | Range in their sensitivity analysis |
| Q_10_ |  | 3.0 | Adjusted to fit measurements | 2.1 – 3.9 | 1.9 – 6.4 | Chapman & Thurlow^67^ | factors based on CO_2_ emission |

* 'default' in this column means parameterization used for modelling fluxes in a boreal fen site (Degerö Stormyr, Sweden).
